# Electrochemical Detection of Dopamine Using 3D Porous Graphene Oxide/Gold Nanoparticle Composites

**DOI:** 10.3390/s17040861

**Published:** 2017-04-14

**Authors:** Sung-Sik Choo, Ee-Seul Kang, Inbeom Song, Donghyun Lee, Jeong-Woo Choi, Tae-Hyung Kim

**Affiliations:** 1 School of Integrative Engineering, Chung-Ang University, Seoul 06974, Korea; sschoo0476@naver.com (S.-S.C.); eeseul94@naver.com (E.-S.K.); sk8nscore@gmail.com (I.S.); dhlee@cau.ac.kr (D.L.); 2 Department of Chemical and Biomolecular Engineering, Sogang University, Seoul 04109, Korea; jwchoi@sogang.ac.kr

**Keywords:** graphene oxide, porous structure, gold nanoparticles, indium tin oxide, neurotransmitters, dopamine, composites, electrochemical detection

## Abstract

The detection of dopamine in a highly sensitive and selective manner is crucial for the early diagnosis of a number of neurological diseases/disorders. Here, a report on a new platform for the electrochemical detection of dopamine with a considerable accuracy that comprises a 3D porous graphene oxide (pGO)/gold nanoparticle (GNP)/pGO composite-modified indium tin oxide (ITO) is presented. The pGO was first synthesized and purified by ultrasonication and centrifugation, and it was then further functionalized on the surface of a GNP-immobilized ITO electrode. Remarkably, owing to the synergistic effects of the pGO and GNPs, the 3D pGO-GNP-pGO-modified ITO electrode showed a superior dopamine-detection performance compared with the other pGO- or GNP-modified ITO electrodes. The linear range of the newly developed sensing platform is from 0.1 μM to 30 μM with a limit of detection (LOD) of 1.28 μM, which is more precise than the other previously reported GO-functionalized electrodes. Moreover, the 3D pGO-GNP-pGO-modified ITO electrodes maintained their detection capability even in the presence of several interfering molecules (e.g., ascorbic acid, glucose). The proposed platform of the 3D pGO-GNP-pGO-modified ITO electrode could therefore serve as a competent candidate for the development of a dopamine-sensing platform that is potentially applicable for the early diagnosis of various neurological diseases/disorders.

## 1. Introduction

Dopamine is a part of the catecholamine family that is produced by the dopaminergic neurons in the brain [1]. Dopamine is known as a critical signal-transmission element between the neurons since it is affiliated with most of the important human body function such as motor control, reward, motivation, and cognitive functions [2,3,4,5]. Owing to this importance of dopamine, it has been reported that insufficient dopamine levels in the blood, or the loss of dopaminergic neurons in the brain, could result in a number of severe neurological diseases such as Parkinson’s disease, drug addiction, psychosis, and attention deficit hyperactivity disorder (ADHD) [6,7]. To address this issue, numerous studies have reported various methods for the detection of dopamine in a highly sensitive and selective manner, which could be readily utilized for the early diagnosis of dopamine-related neurological diseases [8,9,10].

Among the currently available methods (e.g., ELISA, colorimetric methods, Raman, HPLC) [11,12,13], the electrochemical-detection technique is considered one of the most efficacious tools for dopamine detection, owing to its convenience, rapid detection time, and cost effectiveness [14]. Dopamine is a redox-active material that could be reduced or oxidized at specific known electrical potentials, and its electrical property could be used as an indicator to detect the presence of dopamine in a sample (usually human blood). However, one critical barrier in the use of such an electrochemical dopamine-sensing method is the signal interference from other biological molecules (e.g., uric acid (UA), ascorbic acid (AA), catecholamine molecules). The signal interference could significantly deteriorate the sensitivity of the dopamine detection [15,16,17], because the reduction and oxidation potentials of these biological molecules reportedly overlap with those of dopamine [18]. In addition, the electrochemical sensitivity of dopamine is still lower than those of the other conventional methods such as HPLC and ELISA, which remains as a significant impediment to overcome prior to the practical use of this method for precise dopamine detection.

To this end, numerous attempts have been made to overcome the selectivity and sensitivity issues by functionalizing the surface of the electrodes or integrating other types of conductive materials. Graphene, composed of pure carbon molecules that are arranged in a two-dimensional (2D) honeycomb structure, has been widely applied for various research areas, such as batteries, display panels, solar cells, and even biomedical applications [19,20,21,22]; furthermore, the noticeable dopamine-detection performances of graphene derivatives have been reported [23], and these are mostly owing to the π–π and electrostatic interactions between the surfaces of the graphene oxides (GOs) and the dopamine molecules [24]. Various grapheme-derivative-modified electrodes including graphene/glassy carbon electrode (GCE), graphene–gold nanoparticles/GCE, TiO_2_–graphene/GCE, and GO/GCE electrodes have been developed to enhance the performances of dopamine biosensors [25,26,27]. However, porous graphene oxide (pGO), a type of grapheme-oxide sheet with copious hydroxyl groups and a porous surface [28], was discovered and could enhance the electrostatic interaction between the pGO and the analytes, while also facilitating the electron transfer between the molecules and the underlying electrode substrates [29]. Despite its promises in a wide spectrum of applications, the use of pGO for biosensor applications, especially for the electrochemical detection of dopamine, has not been reported. A three-dimensional (3D) pGO/Gold nanoparticle-modified indium tin oxide (ITO) electrode that is effective for the electrochemical detection of dopamine is therefore reported here. The pGO was synthesized through harsh ultra-sonication and centrifugation, and was further embedded on the surface of both gold nanoparticles (GNPs) and ITO surfaces. Subsequently, the pGO structure and the pGO-GNP-pGO composites on the ITO electrodes were individually characterized, and this was followed by the testing and comparing of the dopamine-detection performance of each electrode. Thereafter, the ITO/pGO–GNP–pGO composite, which showed the best electrochemical redox signals in the presence of dopamine, was utilized to achieve linear correlations between the peak intensities and the dopamine concentrations. Lastly, the selectivity of the developed sensor toward dopamine detection was confirmed using ascorbic acid and dextrose as the interfering molecules.

## 2. Materials and Methods

### 2.1. Materials

The ITO electrode was obtained from U.I.D. (Cheongju, Korea), and the gold nanoparticles with a diameter of 60 nm were purchased from BB Solutions (Cardiff, UK). The single-layer graphene oxide (275 mg/L) that was dispersed in water was acquired from Graphene Supermarket (Calverton, NY, USA), and polydimethylsiloxane (PDMS) was obtained from Dow Corning Corp., (Midland, MI, USA). Lastly, (3-Aminopropyl)triethoxysilane (APTES), Dulbecco’s phosphate-buffered saline (DPBS), cysteamine hydrochloride, dopamine, and the interfering materials used for the selectivity test including the AA and glucose (dextrose) were all purchased from Sigma Aldrich (Saint Louis, MO, USA).

### 2.2. Synthesis of Porous Graphene Oxide

To synthesize the pGO, the GO dissolved in the distilled water (DW) was first sonicated for 30 min to disperse the aggregated GO particles. Thereafter, 20 mL (200 μg/mL) of the sonicated GO solution was subjected to an ultrasonic homogenizer for 12 h using a sharp ultrasonic probe (diameter: 5 mm). Cooling units were not utilized to increase the temperature during the ultrasonication process. The GO solution was then distributed into 1.5 mL conical tubes and centrifuged for 10 min (12,000 rpm). The supernatant was collected and used for TEM imaging and electrochemical studies.

### 2.3. Electrode Preparations

The preparation of the working electrodes involved cleaning the ITO-coated glass (10 Ω/cm^2^, 0.5 mm thickness) through a consecutive sonication process with a 1% Triton X-100 solution, DW, and ethanol. The electrodes then underwent a treatment of oxygen plasma for an effective attachment of the APTES, and this was followed by a 15 min coating with a 5% APTES solution. After the removal of the residual APTES solution with the ethanol, the electrodes were baked for 2 h in an oven at 80 °C for an improved annealing, resulting in stable covalent bond between the –OH groups of ITO electrode and silicon oxide groups. A chamber, however, was needed for the electrode to contain a small volume of the solution for the detection, and such a container was fabricated on the surface of the electrodes using a commercially available circular plastic chamber with a 1 cm diameter and PDMS as an adhesive. Subsequently, the gold nanoparticles were distributed into the chamber, and the electrodes were kept in the refrigerator (4 °C) overnight. The gold nanoparticles were then washed off with DW, and the electrodes were coated with the cysteamine hydrochloride and GO solution for 2 h and 1 h, respectively. As well-known, the thiol group of cysteamine could form stable Au-S bond on the surface of GNPs via self-assembly process, leaving free amine groups that are effective for the immobilization of GNPs. Since ITO substrates were functionalized with APTES for GNP immobilization prior to the cysteamine modification, both the ITO and GNP were positively-charged in the aqueous medium owing to the presence of free amine groups, which could attract negatively-charged pGO via electrostatic interactions. Lastly, the cysteamine hydrochloride and GO solutions were washed off with the DW after the coating process.

### 2.4. Electrochemical Detection

Cyclic voltammetry (CV) and amperometric detection were conducted using a DY2000 Series Multi-Channel Potentiostat (Digi-Ivy). While the ITO working electrodes were used for the dopamine-detection platform, the Ag/AgCl (1 M KCl) and a platinum wire were each used as the reference electrode and the counter electrode, respectively. For the CV, various dopamine-solution concentrations were prepared and placed in the chip chamber for the detection. The scan rate and the range are 0.05 V/s, and −0.1 to 0.6, respectively. Lastly, for the selectivity test, the amperometric detection was completed after 20 μL of the 10 μM dopamine, the AA, and glucose were added to the DPBS-filled electrode chamber. The sampling time, initial voltage and sensitivity are 0.05 s, 0.3 V, and 10^−6^ A, respectively. All of the experiments were conducted at a temperature of 25 °C. In the case of calculation of the active surface area, the Randles-Sevcik equation was used described as,

*i_p_* = (2.69 × 10^5^)n^3/2^AD^1/2^v^1/2^C

where *i_p_* = peak current, n = number of electrons involved, A = electrode area (m^2^), D = diffusion coefficient (m^2^/s), v = scan rate (V/s) and C = concentrations of analytes (mol/L).

### 2.5. Statistical Analysis

The height of the cathodic peaks (Ipc) in the cyclic voltammogram were used for the quantitative analysis. The data were analyzed using the computerized statistical program Origin 8 or Microsoft Excel 2013. Data are expressed as mean ± SE (*N* = 3). The significant differences were determined for *p* < 0.05.

## 3. Results

### 3.1. Structural Characterizations of pGO-GNP-pGO 3D Complex

As described above, graphene is a 2D material with wide applications for various areas including biosensing [30,31], especially for dopamine detection [32]. Although the accessibility of GO is relatively higher than that of graphene, it is almost non-conductive owing to its partially fragmented honeycomb structure and the numerous hydroxyl groups on its surface. However, despite its relatively low conductivity, GO can retain substantial electrochemical detection capabilities either by itself or in combination with other electrocatalytic materials, such as gold, silver, and platinum [33,34].

**Figure 1:**
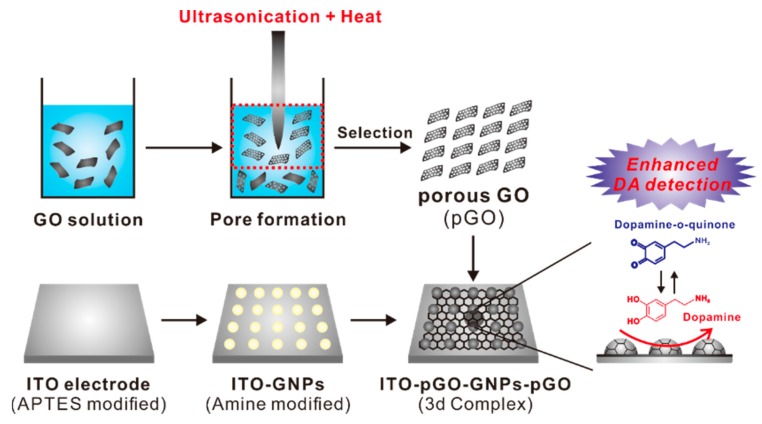
Schematic diagram depicting the detailed steps for the generation of the ITO-porous graphene oxide (pGO)–gold nanoparticle (GNPs)–pGO 3D complex that was used for the electrochemical detection of dopamine.

**Figure 2:**
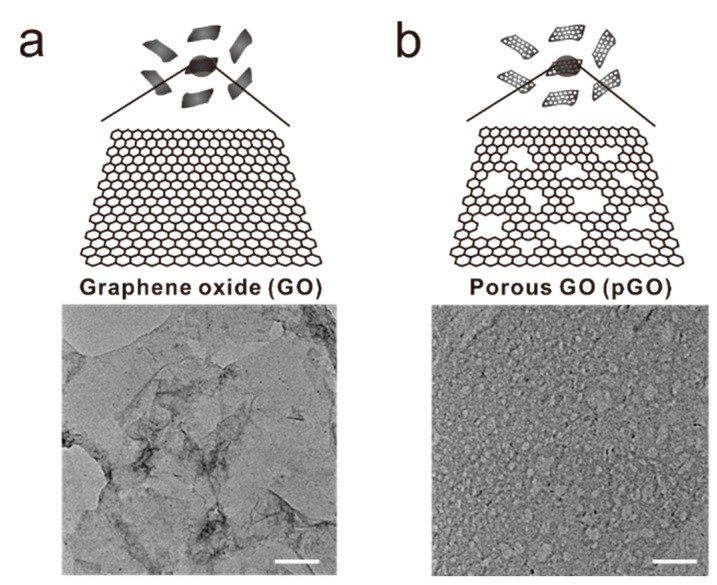
Schematic diagram and transmission electron microscopic images of (**a**) normal graphene oxide sheet and (**b**) porous graphene oxide sheet. Scale bar = 100 nm.

The force that governs the interaction between dopamine and GO has still not been clearly unveiled; however, it is clear that the significant difference between graphene and GO arises from the hydrophilic and negatively charged nature of the GO surface. The surface of pGO is characterized by both abounding amounts of hydroxyl groups and nanosized holes through which the electrons freely pass through. Its surface properties could be exploited extensively to enhance the electrochemical-redox signals of the dopamine molecules. In light of this, the GO solution underwent a series of intense ultrasonic processes without an additional cooling compartment, as shown in Figure 1. The supernatant was collected and centrifuged to achieve a highly porous GO. The pGO was then modified on the GNP-immobilized electrode substrate via an electrostatic interaction to fabricate the ITO-pGO-GNPs-pGO 3D complex. Both the ITO electrode and the GNPs were functionalized with the APTES and cysteamine to give positive charges on their surfaces prior to pGO modifications (Figure 1). The porous structure of generated pGO was confirmed by transmission electron microscope (TEM), as shown in the Figure 2, which revealed a stark difference between the pGO and the regular GO. The size of graphene sheet was found to be around 500 nm–1 μm and the size of the holes on the pGO plane was 10–50 nm.

**Figure 3:**
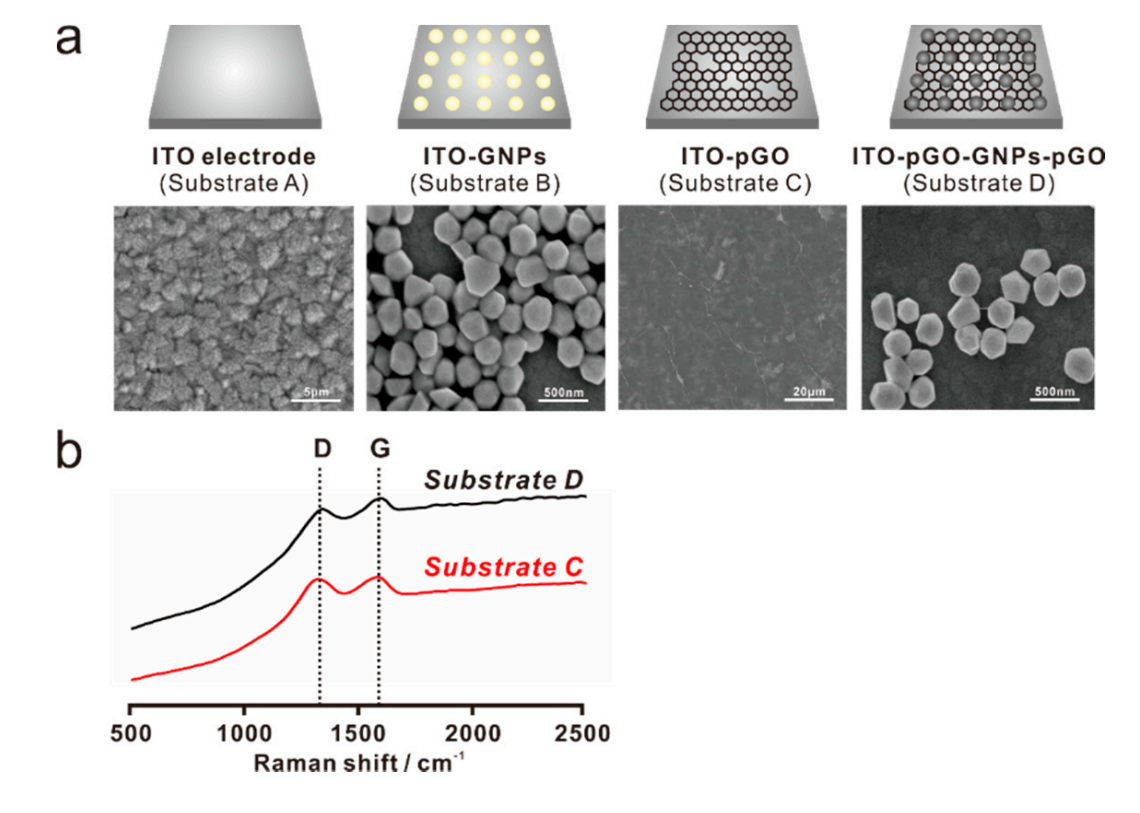
Field emission scanning electron microscopic (FE-SEM) images of the four different substrates: (**a**) bare ITO electrode (Substrate A), gold nanoparticle (GNP)-modified ITO electrode (Substrate B), pGO-modified ITO electrode (Substrate C), and ITO-pGO-GNP-pGO 3D structure (Substrate D); (**b**) Raman spectroscopy of Substrate C and Substrate D, showing the clear D (1350 cm^−1^) and G (1580 cm^−1^) bands of the pGO material.

After the confirmation of pGO structure, four different substrates: ITO electrode (Substrate A), gold nanoparticle-modified ITO electrode (Substrate B), pGO-modified ITO electrode (Substrate C), and ITO-pGO-GNPs-pGO were characterized by scanning electron microscopy and Raman spectroscopy. As shown in Figure 3a, Substrate A showed the typical ITO-deposited topological characteristics, whereas these ITO grains were not present on Substrate C owing to the presence of a pGO sheet on the top of the ITO substrate. In addition, Substrate C showed some wrinkle-like structures, which are the general morphology of the graphene-modified surface; however, the nanosized holes on the surface of the pGO, that were seen in the TEM images (Figure 2b), could not be observed due to the limited resolution of SEM. Substrate B is an ITO electrode where the gold nanoparticles (GNPs) were immobilized via the modifications on the ITO surface. The diameter size of the GNPs is from approximately 200 nm to 300 nm based on the SEM image (Figure 3a). Owing to the presence of the pGO on Substrate B and Substrate D, clear D (1350 cm^−1^) and G (1580 cm^−1^) peaks were observed in both substrates in contrast to substrates A and C. Generally, the G band indicates the in-plain vibrations of SP^2^-bonded carbon atoms, while the D band represents the out-of-plane vibrations (structural defects). The I (D/G) values of Substrate C and Substrate D were calculated as 0.98 and 0.96, respectively, indicating that the pGO structure is similar to that of the GO. Accordingly, the conclusion here states that, based on the TEM and SEM images and the Raman spectroscopic results, the pGO was successfully synthesized and modified on the surface of the ITO-GNP substrates, thereby elucidating its application possibility regarding electrochemical dopamine detection.

### 3.2. Electrochemical Detection of Dopamine Using Different Substrates

**Figure 4:**
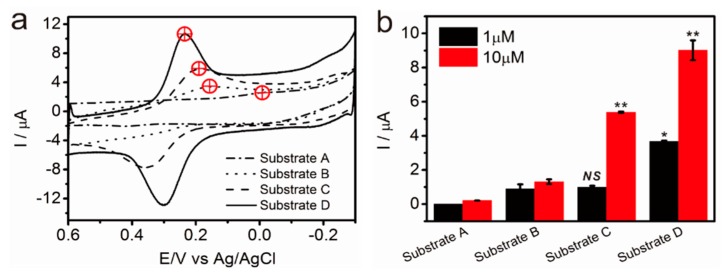
(**a**) Cyclic voltammetry (CV) curves of Substrate A, Substrate B, Substrate C, and Substrate D in the presence of 10 μM of dopamine for which DPBS (pH 7.4) was used as the electrolyte. The red marks represent the reduction potential (vs. Ag/AgCl) that appeared on the voltammogram; (**b**) The peak current intensities of each substrate in the presence of 1 μM or 10 μM of dopamine. (*n* = 3, unpaired student’s *t*-test, * *p* < 0.05, control group: Substrate B with 1 μM of dopamine, ** *p* < 0.01, control group: Substrate B with 10 μM of dopamine).

As previously mentioned, dopamine is vital to several human bodily functions, and numerous studies have reported effective ways to detect dopamine with a considerable accuracy. However, owing to the distinct nature of dopamine that reduces or oxidizes at specific electrical potentials, electrochemical tools have gained significant attention for their capability to detect dopamine in a simple, rapid, and sensitive manner. After the confirmation of pGO structure as discussed in Section 3.1, we first compared the electrochemical signals that were obtained from four different graphene oxide sheet, including commercially available nano graphene oxide, graphene oxide, pGO-1 and pGO-2. The pGO-1 and pGO-2 were synthesized by varying the time for ultrasonication processes from 6 h (pGO-1) to 12 h (pGO-2). As shown in the Figure S1, the pGO-2 showed the highest redox peaks when compared with that of NGO, GO and pGO-1 in the presence of 10 μM dopamine, indicating that pGO-2 is the best GO material for the development of dopamine sensor. Next, to compare the dopamine-detection sensitivity of the composite materials, four differently-fabricated substrates (substrates A to D) were subjected to the CV in the presence of dopamine using phosphate-buffered saline (PBS; pH 7.4) as an electrolyte. Figure 4a illustrates the different CV curves of each substrate in the presence of 10 μM of dopamine. An analysis of the CV curves shows that the |E_pa_– E_pc_| values (peak-to-peak separation) are 0.47 V, 0.24 V, 0.176 V, and 0.066 V for Substrate A, Substrate B, Substrate C, and Substrate D, respectively, while the calculated I_pa_/I_pc_ values are 1.22, 0.33, 0.96, and 1.03 for Substrate A, Substrate B, Substrate C, and Substrate D, respectively. Consequently, these values indicate that the redox reaction of dopamine on those electrodes was quasi-reversible. Of the four different substrates, however, the dopamine CV curve of Substrate D most resembles the ideal reversible redox reaction (|E_pa_ – E_pc_| = 0, I_pa_/I_pc_ = 1). The I_p_ values of Substrate D in the presence of the 10 μM of dopamine are respectively 42.8, 6.88, and 1.67 times higher than those of Substrate A, Substrate B, and Substrate C, implying that Substrate D is the most suitable candidate for the electrochemical detection of dopamine. Moreover, Substrate D even resulted in a highly enhanced reduction current in the presence of 1 μM of dopamine, while the other substrates either failed to detect the presence of dopamine, or nominally produced insignificant levels of electrochemical signals under the same condition.

The active surface area of the Substrate D was calculated to be 0.2927 cm^2^ based on the voltammogram obtained using K_3_Fe(CN)_6_ as a redox couple and the Randles-Sevcik equation (Figure S2). The results show that the newly developed platform, the pGO-GNP-pGO 3D complex (Substrate D), is the most capable candidate for the chemical detection of dopamine compared with the other types of substrates.

### 3.3. Detection of Dopamine Using pGO-GNP-pGO 3D Complex

One of the most important parameters in the development of a sensing platform is the linearity of the signal intensities and the concentration of the target analyte. By calculating this correlation, the signal intensity, the dopamine-obtained redox peaks, could therefore be translated into the desired-value dopamine concentration. Since the pGO-GNP-pGO 3D complex (Substrate D) showed the best performance in terms of dopamine detection, as confirmed in Section 3.2, a subsequent attempt was enacted to achieve the electrochemical signals with the varying of the dopamine concentrations. Considering the excellence of the developed platform for dopamine detection, the dopamine concentration was varied from the nano-molar range to the micro-molar range.

**Figure 5:**
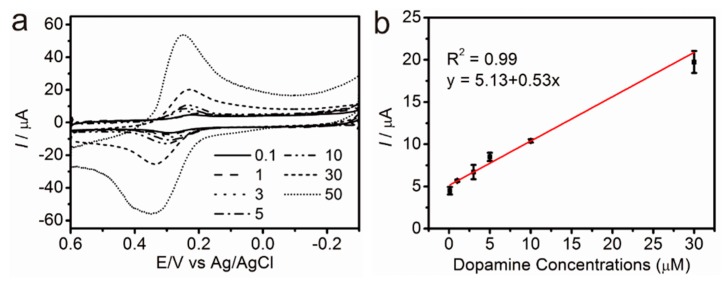
(**a**) CV curves obtained from Substrate D in the presence of dopamine (0.1 μM to 30 μM) and (**b**) linearity of the current values and dopamine concentrations. (*n* = 3, electrolyte: DPBS (pH 7.4)).

Remarkably, even the 0.1 μM of dopamine showed weak redox peaks that were distinguishable with the control group (DPBS without dopamine). The reduction and oxidation peaks on the cyclic voltammogram clearly increased with increasing the dopamine concentrations from 0.1 μM to 50 μM, as shown in Figure 5a. The reduction peak (Epc) is approximately 240 mV ± 10 mV with the dopamine-concentration changes (0.1 μM to 50 μM), proving the signal stability of the developed pGO-based composite for the dopamine detection. The LOD of the developed sensor was calculated as 1.28 μM with the linear range of 0.1 μM to 30 μM, which is superior to or similar to the previously reported sensing platform (Table 1).

**Figure 6:**
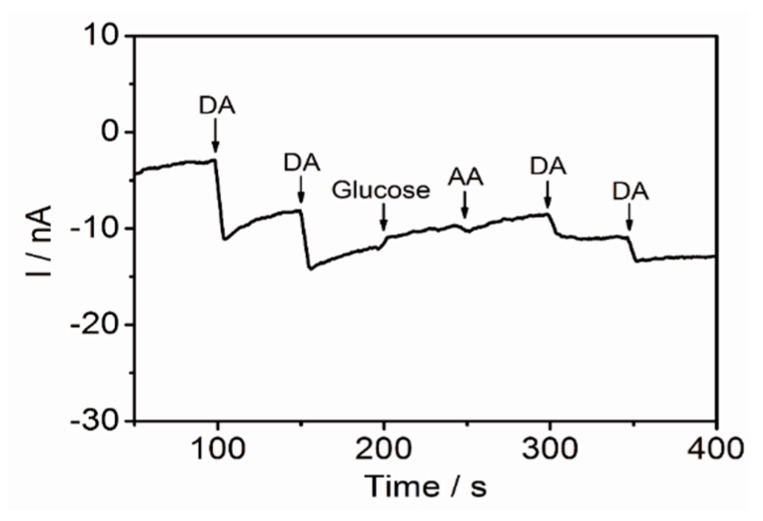
Amperometric detection of 10 μM of dopamine with addition of two different types of interfering molecule (glucose and AA) for which DPBS (100 mM, pH 7.0) is used as an electrolyte. Potential applied = 0.3 V vs. Ag/AgCl.

Since dopamine generally co-exists with the other molecules in the blood plasma including the AA, UA, glucose, and protein, it is important to confirm the selectivity of the developed electrode toward dopamine detection. As an interfering molecule, AA and glucose were chosen for amperometric dopamine detection. As shown in Figure 6, the addition of the dopamine (10 μM) showed significant changes in the current (8.1 nA and 6.0 nA), while the addition of the same concentrations of the AA and the glucose, respectively, did not result in any remarkable signal changes. The signal changes induced by the addition of the DA appeared on the voltammogram, indicating that the developed sensor is excellent in terms of the selectivity toward the dopamine detection. These findings can be used to conclude that the developed platform, the 3D pGO-GNP-pGO composite-modified ITO electrode, is effective for the electrochemical detection of dopamine, one of the most important neurotransmitters, in terms of both sensitivity and selectivity.

## 4. Discussion

Conclusively, the new material reported in this paper, 3D pGO-GNP-pGO, underwent two perceptible alterations in its surface property. First, the graphene oxide substrate was imbued with substantial porosity on its surface (pGO), and the porous surface was further incorporated with GNPs. The new material has proven its suitability for the electrochemical detection of dopamine, and this could be attributed to the changes on the substrate surface that were undertaken in this research, which eventually delivered two benefits: increased specificity and selectivity.

First, the porous nature of the synthesized pGO demonstrated higher efficiency in dopamine detection than that of a typical GO sheet. The enhanced specificity was retained even in low concentrations of dopamine (10 μM). The reduction peaks that were obtained from the 3D pGO-GNP-pGO are 42.8, 6.88, and 1.67 times higher than those of the bare ITO, ITO-pGO, and ITO-GNP electrodes, respectively The superior specificity of the 3D pGO-GNP-pGO substrate for dopamine detection is still unclear, however, but it is reasonable to expect that the presence of many hydroxyl groups on the pGO plane might facilitate hydrogen-bonding formation between the dopamine molecule and the pGO. Furthermore, it could be postulated that the nanoholes on the pGO plane assisted the electron transfer from the dopamine molecules to the GNP surfaces, which could contribute to the enhancement of the electrochemical-redox signals. On the other hand, the ability of the pGO in the detection of dopamine was reportedly further improved by the incorporation of GNPs, which are commonly known to enhance electrochemical-signals. The selectivity of the 3D pGO-GNP-pGO toward the dopamine detection was further confirmed with amperometry analysis in the presence of other interfering molecules including 10 μM of the AA and glucose. However, further studies need to be conducted to thoroughly investigate the physicochemical properties of pGO, especially in terms of the interaction between the pGO and dopamine molecules.

In conclusion, the 3D pGO-GNP-pGO is excellent for dopamine detection and could be utilized for the development of biosensors that are capable of detecting other types of neurotransmitters (e.g., glutamate, acetylcholine, serotonin, gamma-Aminobutyric acid), and these might be helpful for the early diagnosis of various neurological diseases [41].

## Figures and Tables

**Table 1:** Comparison of graphene-modified electrodes used for the detection of dopamine.

Electrode	Methods	Linear Range (μM)	LOD (μM)	Refs.
GO/GCE	CV ^1^, EIS ^2^ DPV ^3^	1–15	0.27	[35]
GR/GCE	CV, DPV	4–100	2.64	[36]
GR-AuNP/GCE	CV, DPV	5–1000	1.86	[37]
Chitosan-GR/GCE	CV, DPV	1–24	1	[38]
TiO2-GR/GCE	CV, DPV	5–200	2	[39]
Au/RGO/GCE	CV, DPV	6.8–41	1.4	[40]
pGO-GNP-pGO	CV, AM ^4^	0.1–30	1.28	This work

^1^ Cyclic voltammetry; ^2^ Electrochemical impedance spectroscopy; ^3^ Differential pulse voltammetry; ^4^ Amperometry.

## Supplementary Materials

The following are available online at http://www.mdpi.com/1424-8220/17/4/861/s1, Figures S1 and S2.
