# Supplementary material for: Electrochemical Detection of Dopamine Using 3D Porous Graphene Oxide/Gold Nanoparticle Composites
Source: Sensors (Basel). 2017 Apr 14;17(4):861. doi: 10.3390/s17040861 (PMC5424738; doi:10.3390/s17040861)
Supplement: Supplementary file 1 [file sensors-17-00861-s001.pdf]

Supplementary figures and figure captions

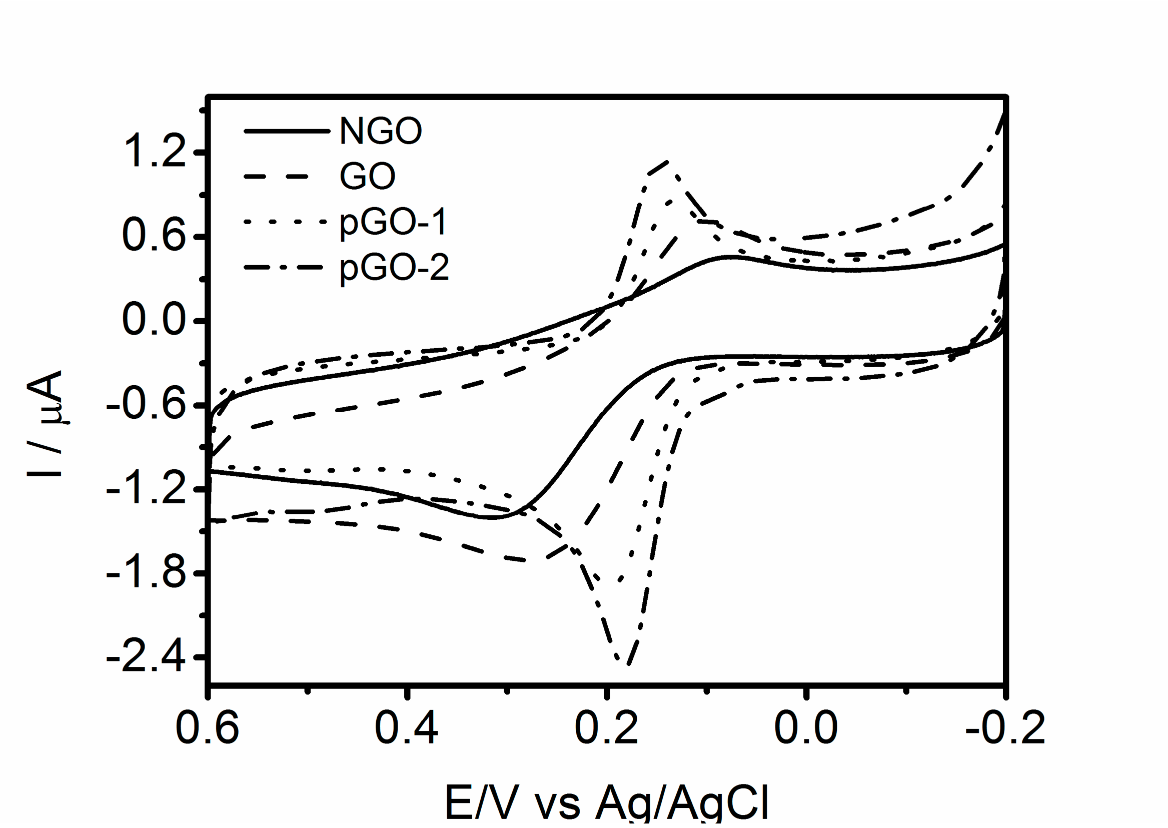

Figure S1. Cyclic voltammetric curves obtained from four different substrates in the presence of 10 μM dopamine using DPBS (pH 7.4) as an electrolyte. Scan rate = 0.05 V/s. 'NGO', 'GO', 'pGO-1' and 'pGO-2'stand for 'commercial nano graphene oxide', 'graphene oxide', 'porous graphene oxide synthesized by 6 hours of ultrasonication and 'porous graphene oxide synthesized by 12 hours of ultrasonication'.

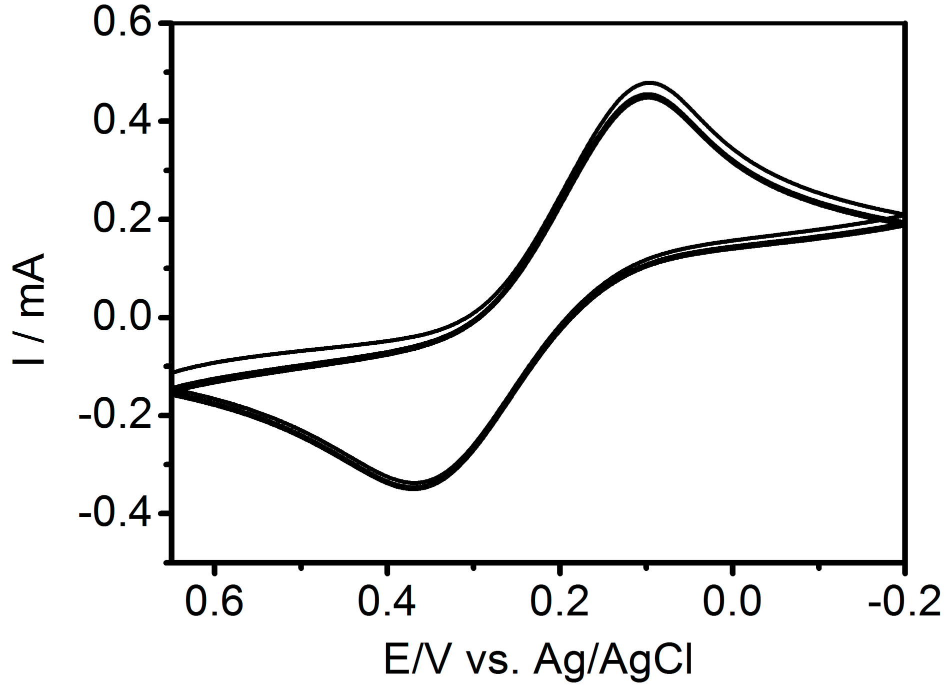

Figure S2. Cyclic voltammetric curves obtained from Substrate D using 10mM $K_3Fe(CN)_6$ in 1M KNO_3 as an electrolyte for the calculation of active surface area of the electrode. Scan rate = 0.05 V/s.
